# Dynamic characteristics and evolution laws of underground brine in Mahai salt lake of Qaidam Basin during mining process

**DOI:** 10.1038/s41598-024-61196-y

**Published:** 2024-05-11

**Authors:** Zhihan Kong, Guangcai Wang, Qingyu Li, Quansheng Zhao, Shuya Hu

**Affiliations:** School of Environmental Science and Engineering, Qingdao University, Qingdao, 266100 Shandong China

**Keywords:** Qaidam Basin, Salt Lake, Underground brine, Rock salt medium, Dissolution mining Calcium ion, Environmental impact, Environmental sciences, Hydrology

## Abstract

In the late stage of underground brine mining in salt lakes, the method of injecting fresh water is often used to extract the salt from the brine storage medium. This method of freshwater displacement breaks the original water–rock equilibrium and changes the evolution process of the original underground brine. To explore the mechanism of salt release in saline water-bearing media under conditions of relatively fresh lake water dissolution, this paper analyzes the changes in the chemical parameters of brine from 168 sampling points in the Mahai salt lake in the Qaidam Basin at three stages (before exploitation, during exploitation, and late exploitation) by correlation analysis, ion ratio analysis, and other methods and investigate the variations in porosity and the evolution laws of brine. The results show that the changes in the main ion content and brine mineralization during the exploitation process are small. The changes in Ca^2+^ content are significant due to the low solubility of calcium minerals, the precipitation of gypsum during the mixing process, and the adsorption of cations by alternating with Ca^2+^. Primary intergranular pore skeletons are easily corroded to form secondary pores, which increase the geological porosity. Na^+^ and Cl^-^ are the dominant ions in the brine in the study area, but the concentration of Ca2 + decreased significantly under the influence of mining, by 41.7% in the middle period and 24.5% in the late period. The correlation between Ca^2+^ and salinity changes significantly in different mining stages, and the reason for the decrease of Ca^2+^ may be due to the influence of mineral dissolution, mixing, and anion-cation exchange. The porosity of the layer in the study area showed the opposite trend of Ca^2+^, and the porosity increased first and then decreased. The innovation of this paper lies in analyzing the reasons and mechanisms of the disturbance of artificial dissolution mining on stratum structure by comparing the hydrochemical characteristics and porosity of underground brine storage media in three different mining stages. The research in this paper provides a theoretical basis for the calculation of brine resource reserves and the sustainable development of underground brine in salt lake areas.

## Introduction

Underground brine is a liquid mineral resource that is widely recognized^1^. It can be used for development and utilization in industries such as industry and agriculture. Underground brine has a high salinity and contains many important elements, making it an important source of chemical raw materials such as potassium, bromine, iodine, lithium, and boron. China has large reserves of potassium salts in modern saline lakes such as Qaidam Basin in Qinghai Province and Lop Nur in Xinjiang, of which liquid brine mines are the most abundant^2–5^. The underground brine in Qaidam Basin is intercrystalline brine stored in salt rock medium, whose medium skeleton has strong solubility. Underground brine contains characteristics such as high mineralization, multiple components, and easy precipitation^6,7^. In the natural state, the brine migration rate in the brine-containing layer is very slow, and the rock salt pore medium and brine are in a relatively stable chemical equilibrium state^8–11^. In the mining process, the shallow brine is easy to drain, and the method of injecting fresh water to dissolve and extract brine resources is often used to obtain more brine resources^12^. Freshwater dissolution will break the original water–rock balance and change the brine evolution process. Salt dissolution-salt precipitation in brine-bearing layers not only changes the pore structure and structural features of the medium but also affects the concentration of brine, which will affect the hydrogeological parameters such as porosity, water supply degree, and permeability coefficient of brine reservoir^13^. Therefore, the study of brine hydrochemical dynamics and its evolution law has important scientific guiding significance for the industrial extraction of brine, as well as the calculation and evaluation of resource reserves^14–16^.

Domestic and foreign scholars have conducted extensive research on the hydrochemical characteristics and genesis of groundwater brine. Bo Y et al.^17^ studied the hydrochemical characteristics and controlling factors of salt spring water in the Tarim Basin of western China, and found that the composition of river water in the Tarim Basin is closely related to the surrounding rock types and strong evaporation effects. At the same time, thermo-hydrodynamic calcium halide plays an important role in the hydrochemical evolution of the Tarim Basin. Yu et al.^18^ used the dynamic monitoring data of underground brine in the first mining area of Qarhan. He et al.^19^ origin and evolution of Li-rich brine in Qaidam Basin.

Salt Lake to establish an evaluation and prediction model for the water quality and quantity of underground brine. Fan et al.^20^ stablished a numerical model of hydrogeology for the saline-water layer in the Tenglong Platform of Lop Nur, Xinjiang, studying the hydrogeological characteristics and migration laws of underground brine under special buried conditions. Yang et al.^21^ used isotope tracing technology to measure the percolation coefficient and specific yield of the Xitang Saline Lake in the Qaidam Basin. Xu et al.^22^ used the audio magnetotelluric method to conduct hydrogeological exploration in the Qaidam Basin, established a model to analyze the distribution and characteristics of groundwater aquifers, and studied the hydrological characteristics of groundwater aquifers. Zhao Quansheng^6^ used mathematical statistics to analyze the distribution changes of the water content in the saline lake brine layer of the Qaidam Basin's Mahai Saline Lake. The water content was influenced by the saline lake's sedimentary environment and presented a changing pattern of small-large-small-large from bottom to top. Zhao et al.^23^ studied the underground brine samples of Mahai Salt Lake in the Qaidam Basin during the initial stage, middle stage, and late stage of mining. Through statistical analysis, correlation analysis, and other methods, they studied the dynamic characteristics of the water chemistry of underground brine in Mahai Salt Lake during mining and analyzed the evolution law of brine. Hu et al.^24^ determined the permeability coefficient of the saline lake brine layer in Mahai Salt Lake in the Qaidam Basin through a pumping experiment, and divided the experimental area into different underground brine enrichment sections. Zhao et al.^25^ used integrated geophysical information methods to detect the rich brine area in Mahai Salt Lake, constructed a geophysical model of underground brine, and detected the saline lake's brine control structure and rich brine layer distribution.

Most relevant studies have focused on the control of diagenesis on the isotope composition and chemical composition of brine, while there is less research on the dynamic characteristics and evolution laws of brine water chemistry during the mining process, especially in terms of understanding the evolution law of underground brine and the release mechanism of salt under conditions of freshwater displacement. This paper takes Mahai Salt Lake in Qaidam Basin as the research object, selects underground brine in its original state (before mining), mid-term mining, and dissolution and dissolution extraction stage (late-stage mining), through ion concentration analysis and correlation analysis, this paper analyzes the change mechanism of calcium ion content in the mining process and the change of porosity caused by the change of calcium ion content, analyzes the evolution law of groundwater, and discusses the influence of solution mining brine on the salt release of groundwater saline aqueous medium, to provide scientific basis for the rational development and utilization of underground brine. This article studies the disturbance of mining on underground brine from the perspective of calcium ion concentration changes, and delves into mechanisms such as mineral dissolution, mixing effects, and cation exchange, studying the brine evolution process from a new perspective. In addition, the introduction of porosity changes is used to analyze the process of brine release from the brine storage medium during solution mining, and multi-scale analysis is used to investigate the dynamic characteristics and evolution laws of underground brine during the mining process. By studying the mechanism of salt release of underground brine storage medium under mining conditions, this paper provides a theoretical basis for the mining of underground brine in salt lakes, as well as scientific evidence for the calculation of water dissolution mining volume and the sustainable development of underground brine.

## Research area

**Figure 1:**
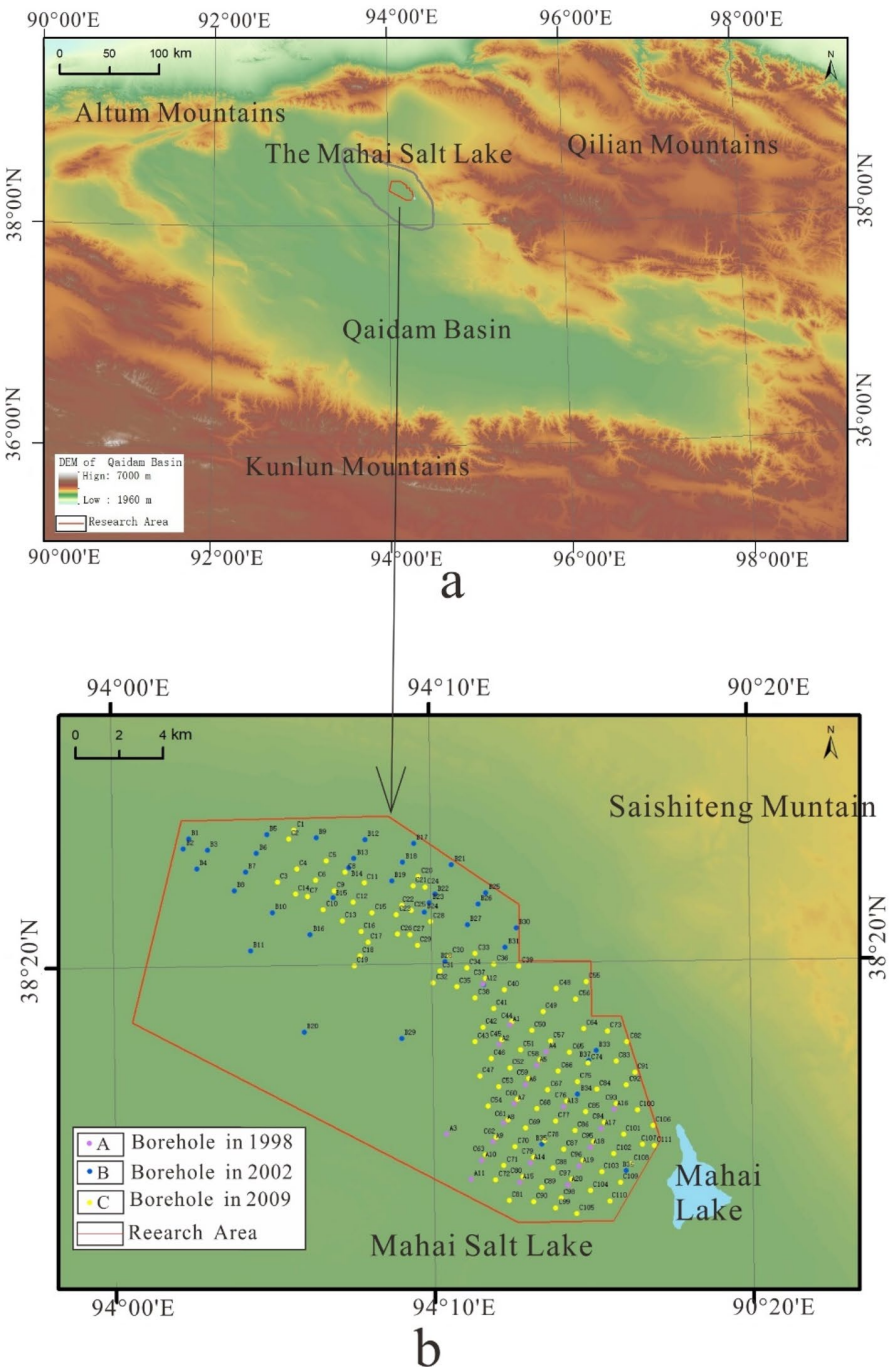
Location of the research area. (**a**) is the Study area location. (**b**) is the Borehole map. (The image is derived from open source information: images generated from LANDSAT public DEM data in the Geospatial Data Cloud).

Mahai Basin, located in the northern Qaidam Basin (Fig. 1), is a sub-basin formed by folding and fault tectonic movements, and the study area is Mahai Salt Lake in the east of the basin. The area is a typical modern inland saline lake sedimentary plain with an elevation of 2,743–2,750 m. The average annual temperature is 2.1°C, the average annual precipitation is 29.61 mm, and the average annual evaporation is 3,040.00 mm The study area belongs to a typical inland arid climate.

The salt sediments in the study area constitute a brine layer and a weak water-permeable layer composed of clay and sand layers. Groundwater is mainly intercrystalline brine with large resource reserves. The natural depth of the brine water level is between 0.75 and 2.13 m, and the salinity is between 307.72 and 463.50 g/L, with an average of 356.90 g/L. Mahai Lake in the southeast of the study area is the water source of the solution extraction stage, and the salinity of the lake is about 95.5 g/L^6,26^. The lake basins contain quaternary unconsolidated deposits and chemical salt deposits in the study area. Moreover, the surface salt crust is typically rough, primarily composed of a blend of fine-grained halite crystals and mud. Quaternary strata, including material from the Pleistocene, Upper Pleistocene, and Holocene series, are extensively distributed across the study area, with exposed layers at the surface. Material from the Lower Pleistocene series remains unexposed. Additionally, the halide layer primarily comprises minerals such as rock salt (NaCl), sodium carnallite, gypsum (CaSO_4_·2H_2_0), calcite (CaCO_3_), feldspar (KAISi_3_0_8_-NaAlSi_3_0_8_-CaAl_2_Si_2_0_8_), sylvine (KCl), and dolomite (CaMg(CO_3_)_2_). In the study area, a large distribution area of quaternary loose deposits and chemical salt sediments are deposited, and two types of loose rock pore water and chemical salt intercrystalline water are abundant.

## Data and methods

Since 2000, the shallow brine of Mahai Salt Lake has been continuously extracted. By 2009, a large-scale falling cone phenomenon occurred in the water level of the brine submersible aquifer. To study the dynamic characteristics of groundwater chemistry during the mining process, we selected and analyzed the water chemistry data from three periods: the early stage of shallow brine extraction (1998), the mid-term of shallow brine extraction (2002), and the later stage of shallow brine extraction (2009). There were 20, 37, and 111 water chemistry data points for each of the three stages, with a total of 168 water chemistry data points. The distribution of research points is shown in Fig. 1b. The pre-mining points are distributed in the southeast of the study area, the mid-mining points are distributed in the north of the study area, and the late mining points are evenly distributed in the north and east of the study area. The western part of the study area is difficult to mine due to topographic factors.

The chemical analysis of brine water was completed by the Testing Center of Comprehensive Geological Mineral Resources Survey Institute of Qaidam, Qinghai Province. The pH value was measured by a portable pH meter, and the specific gravity (relative density) of the brine was measured by a Baume hydrometer^27^. The ion content was determined according to the Analysis Method of Salt and Brine Water. The content of K^+^ and Na^+^ was determined by flame spectrophotometry, the content of Ca^2+^ and Mg^2+^ was determined by the EDTA complex titration method, the content of Cl^-^ was determined by silver nitrate volumetric method, the content of SO_4_^2-^ was determined by barium chloride titration method, and the total dissolved solids (TDS) were the sum of ion mass concentration. Before analyzing the data, the reliability of the data was tested through the cation–anion balance equation, and the error of abnormal values was removed by drawing box plots to ensure the accuracy of the analyzed data. The porosity of the drill core was determined using the weight method.

**Figure 2:**
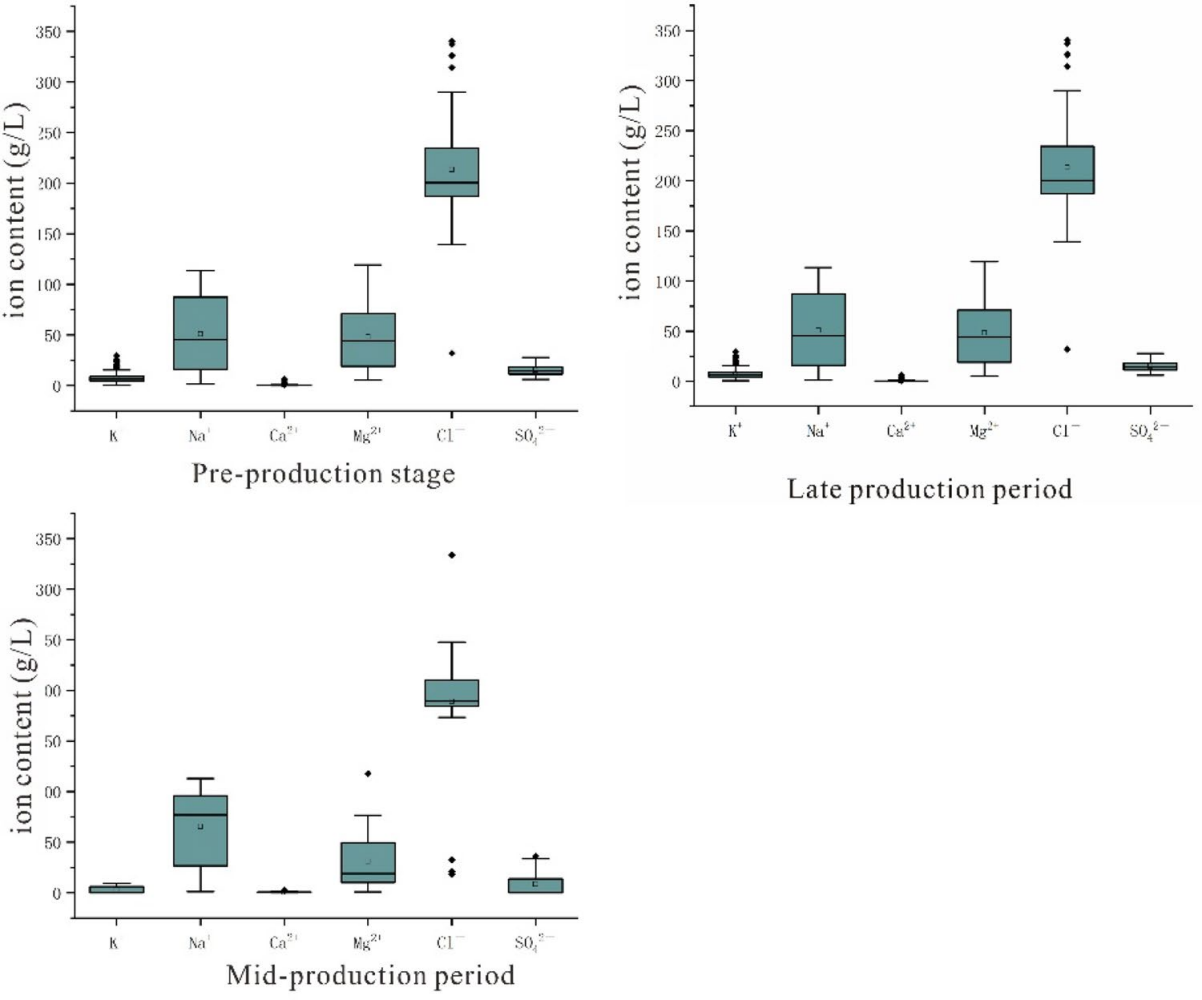
The box plot constructed from the test data of the brine samples.

The anion and cation balance equation was then employed to control the data quality and remove the exception data through the preparation of a box plot. Quality control of the chemical analyses was conducted by analyzing blank samples, carrying out the various measurements in duplicate, and calculating the charge balances. Using the equation indicated below, the charge balance errors for the analyses carried out were found to be < 5%^28,29^.

$$E=\frac{\Sigma {m}_{c}-{\Sigma }_{{m}_{a}}}{\Sigma {m}_{c}+{\Sigma }_{ma}}\times 100$$

where E is the charge balance error (%), m_a_ is the anionic mg equivalent, and m_c_ is the cationic mg equivalent. The charge balance error inferred from the cation/anion balance was < 5%, which was considered an acceptable error for interpretation. A box plot (Fig. 2) was constructed to permit the rejection of outlier values, due to the superiority of this system for application in the case of large amounts of geochemical data^30^. Following the rejection of the outlier values by the box plot using Origin software, values within the normal scope were obtained to give standard analytical conditions. Table 1 shows the hydrochemical statistical characteristic values and porosity data of brine samples in the three stages after passing the reliability test and excluding the abnormal values. Based on the chemical parameters and porosity data of the shallow brine in the study area plot the figure of Changes of Ca^2+^ concentration distribution in different stages, Kriging interpolation method was used to interpolate the Ca^2+^ content and porosity distribution in three periods, which were plotted in Scatter diagrams of SO_4_^2-^ and Ca^2+^ in brine at different stages.

**Table 1:** Hydrochemical statistics of brine in Mahai Salt Lake.

	K^+^ (g/L)	Na^+^ (g/L)	Ca^2+^ (g/L)	Mg^2+^ (g/L)	Cl^-^(g/L)	SO_4_^2-^ (g/L)	pH	density	salinity	Porosity (%)
								(g/cm^3^)		
**Stage 1**			Number of samples added: 20							
Max	9.90	108.00	1.28	29.48	221.70	26.79	8.58	1.24	361.9	19.04
Minimum	4.48	45.50	0.20	9.57	178.10	9.96	7.81	1.21	320.7	11.17
Average	5.76	81.73	0.84	13.80	187.72	15.62	8.28	1.22	334.76	15.28
Standard deviation	2.04	19.97	0.27	5.61	11.09	3.55	0.19	0.01	12.97	2.67
Coefficient of variation	0.35	0.24	0.32	0.41	0.06	0.23	0.02	0.01	0.01	0.17
**Stage 2**				Number of samples added: 37						
Max	9.26	113.00	0.91	117.70	333.70	36.07	8.72	1.33	461.9	28.51
Minimum	5.06	10.35	0.21	6.64	173.40	7.71	7.71	1.02	288.1	14.66
Average	6.83	68.87	0.49	37.49	203.21	8.27	8.27	1.21	326.51	20.60
Standard deviation	1.21	29.35	0.20	27.03	30.70	9.21	0.27	0.05	29.17	3.98
Coefficient of variation	0.18	0.43	0.41	0.72	0.15	0.51	0.03	0.04	0.09	0.19
**Stage 3**				Number of samples added: 111						
Max	15.34	113.70	1.53	94.31	340.50	27.79	9.68	1.46	463.50	25.86
Minimum	0.59	21.65	0.28	9.97	139.30	6.04	7.80	1.27	300.60	12.28
Average	6.44	67.55	0.37	50.83	215.15	15.22	8.69	1.33	335.22	18.79
Standard deviation	2.88	27.08	0.31	21.16	38.91	5.26	0.28	0.02	26.29	2.50
Coefficient of variation	0.44	0.40	0.83	0.42	0.18	0.35	0.03	0.01	0.07	0.13

## Results with discussion

### Analysis of water chemical characteristics

It can be seen from Table 1 that the main ions in the brine samples at the three stages of Mahai Salt Lake have the same order of abundance. The decreasing order of cation concentration is Na^+^ > Mg^2+^ > K^+^ > Ca^2+^, and the average ion concentration is 72.72 g/L、34.04 g/L、6.34 g/L、0.57 g/L, respectively. Among anions, the concentration of Cl^−^ is higher than that of SO_4_^2-^, with average concentrations of 202.03 g/L and 13.04 g/L, respectively. The average and standard deviation of Na^+^ and Cl^−^ in the brine sample are relatively large, and the coefficient of variation is relatively small, indicating that their absolute content in the brine is large, but their relative content does not change much, making them the main ions in the brine. The coefficients of variation of Ca^2+^、K^+^、Mg^2+^、and SO_4_^2−^ are relatively large, indicating that their content changes significantly in the brine, which indicates that they fluctuate significantly with environmental factors. Especially for Ca^2+^, the coefficient of variation changes significantly, indicating that its content is significantly affected by environmental changes.

**Figure 3:**
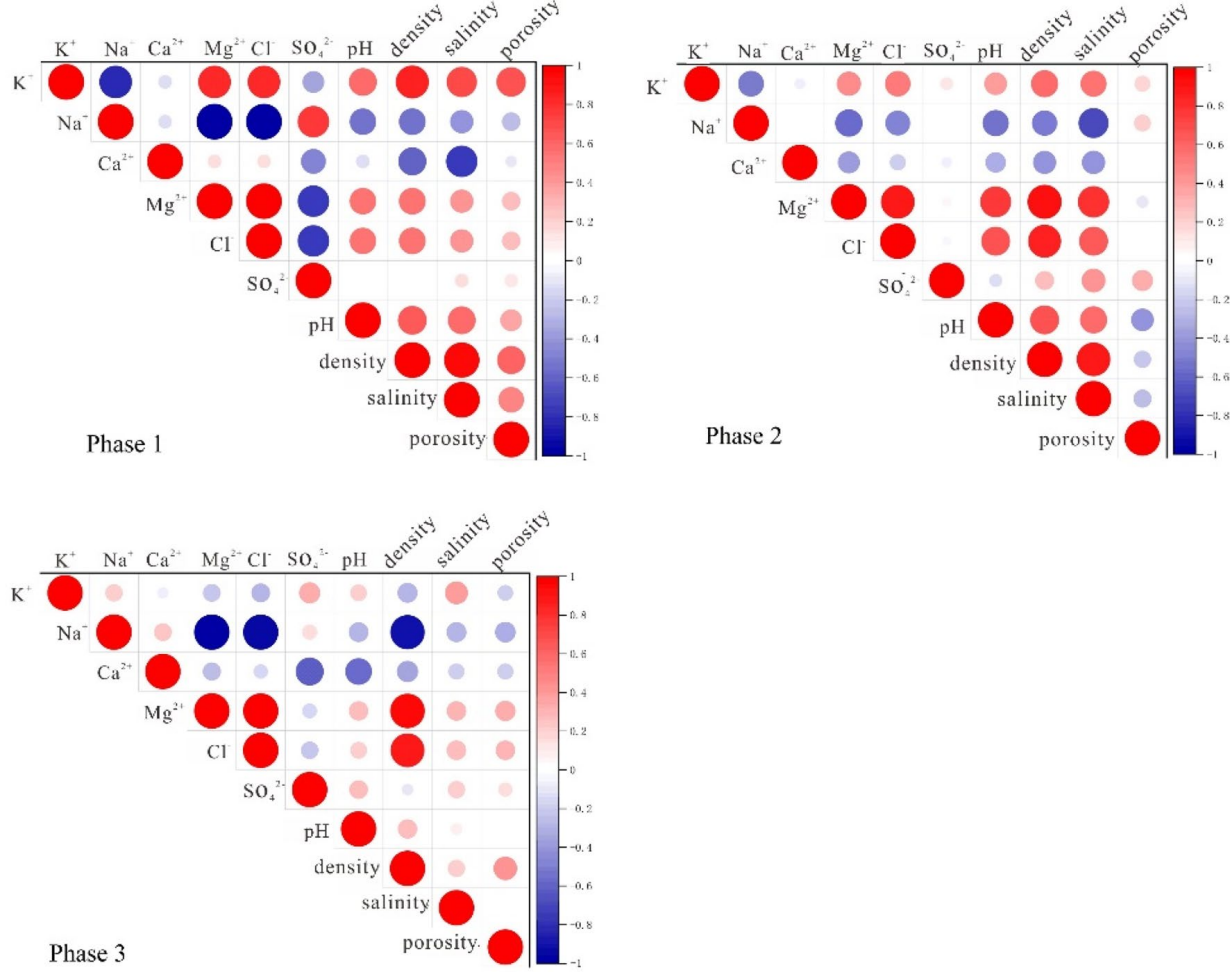
Heat map of correlation analysis.

Mineralization refers to the concentration of dissolved minerals in the brine and is an important index to measure the salt content in the brine. The average mineralization of underground brine in three stages is 334.76 g/L, 326.51 g/L, and 335.22 g/L, respectively. The mineralization of Mahai Lake water (95.50 g/L) injected by solution mining is much lower than that of the primary underground brine. Spearman correlation analysis of main ions and salinity in the brine of Mahai Salt Lake (Fig. 3) shows that in the non-mining period, the Spearman correlation coefficient between Ca^2+^ and salinity is 0.645, showing a significant negative correlation. After mining, the correlation between Ca^2+^ and salinity changed significantly, and the correlation coefficient changed from − 0.64 in 1998 to − 0.42 in 2002 and − 0.26 in 2009, indicating that the correlation between Ca^2+^ and salinity was not obvious. This also indicates that the correlation between Ca^2+^ and mineralization in primary brine is strong, and Ca^2+^ is gradually enriched during the evaporation and concentration process of brine. The disturbance of solution mining has made the correlation between Ca^2+^ and mineralization in secondary brine not significant.

### Characteristics of Ca^2+^ concentration change in the study area

**Figure 4:**
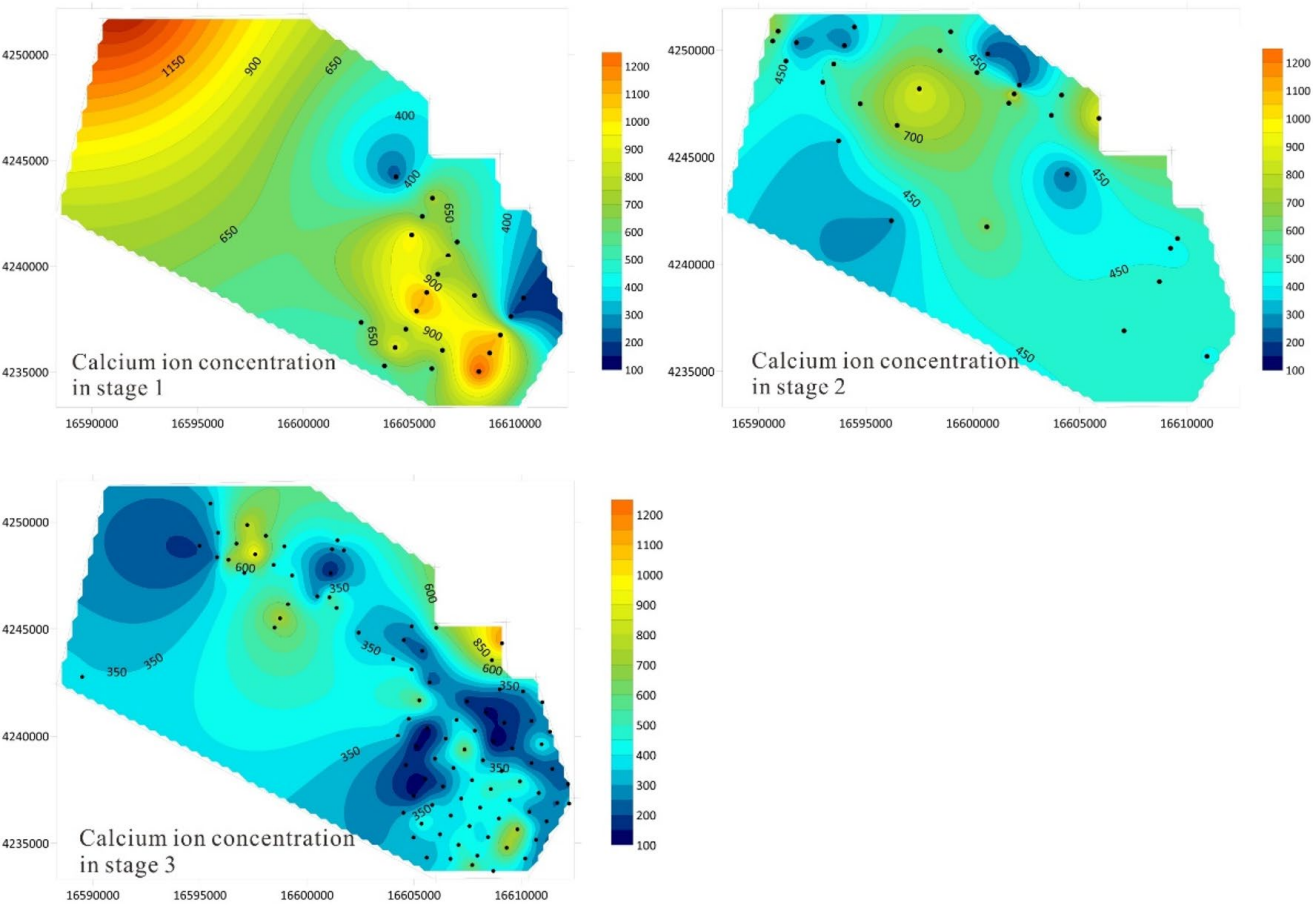
Changes of Ca^2+^ concentration distribution in different stages.

The correlation between Ca^2+^ and mineralization changed significantly in different stages, and Ca^2+^ is sensitive to changes in the water environment. Therefore, Ca^2+^ can be used as a key index to study the salt dissolution and salting-out effect of the brine storage medium. Figure 3 shows the changes in Ca^2+^ concentration distribution in different stages of the study area. It can be seen from the figure that in the early stage of underground brine mining, the concentration of Ca^2+^ in the brine of the study area was relatively high, and the content of Ca^2+^ in most areas exceeded 500.00 mg/L (Fig. 4). The brine with Ca^2+^ concentration exceeding 800 mg/L was distributed continuously in the northwest and southeast of the study area. During the mid-term of mining, the Ca^2+^ concentration decreased significantly, with only a small area in the northwest having a concentration exceeding 600.00 mg/L. Most areas had a Ca^2+^ concentration of 300.00–500.00 mg/L. In the later stages of mining, In the later stage of mining, the Ca^2+^ concentration in the study area was generally lower than 300 mg/L, with only scattered spots exceeding 600.00 mg/L.

During the natural evolution of groundwater, as the mineralization degree increases, the carbonate weathering effect weakens, while the dissolution and evaporation concentration effect of gypsum enhances, which can increase the concentration of calcium ions^31^. During the mining process, the overall calcium ion concentration of the underground brine in the study area shows a decreasing trend, which is the opposite trend of normal groundwater evolution.

## Mechanism analysis of Ca^2+^ concentration change

### Mineral dissolution

**Figure 5:**
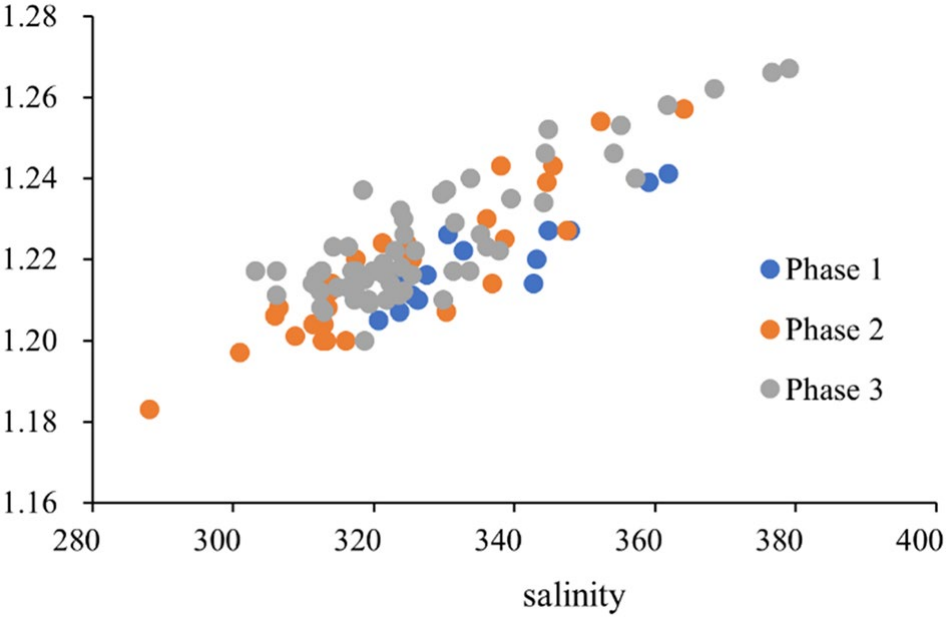
Scatter plot of salinity and density of brine at different stages.

The calcium ion concentration decreased significantly, which may be due to the mixing effect of introducing lighter lake water. The mineralization degree of the water in Mahai Lake is about 95.50 g/L, which is significantly different from that of the primary underground brine. Lighter lake water will dilute the primary underground brine, which may reduce the mineralization degree of the secondary brine, resulting in lower ion concentration. However, according to the scatter plot of mineralization degree and density at different stages (Fig. 5), it can be found that there is a good linear relationship between mineralization degree and density, and the changes in mineralization degree and density of the brine at the three stages are not significant. While diluting the primary underground brine, the lake water also dissolves the minerals in the salt rock reservoir, and the dissolution of the salt rock minerals maintains a high density and mineralization degree of the secondary brine. However, the contribution of calcium ion content to this high mineralization degree has significantly decreased.

The main salt minerals of the storage brine medium in the study area are halite (NaCl), gypsum (CaSO_4_·2H_2_O), Glauberite (Na_2_Mg(SO_4_) _13_·15H_2_O), Sylvinite (KCl), and blondie (Na_2_Mg(SO_4_)_13_·15H_2_O)(Zhao et al., 2017). halite (NaCl) Sylvinite (KCl) and other salts have high solubility and are easily soluble in water. The dissolution of these salt rocks makes the mineralization degree of the secondary brine and the primary brine relatively small. However, gypsum (CaSO_4_·2H_2_O), and Glauberite (Na_2_Mg(SO_4_)_13_·15H_2_O) have relatively low solubility, resulting in a decrease in Ca^2+^ content in the secondary brine. Therefore, the low solubility of calcium-containing minerals is the main reason for the decrease of Ca^2+^ content in the secondary brine.

### Mixing effect

**Figure 6:**
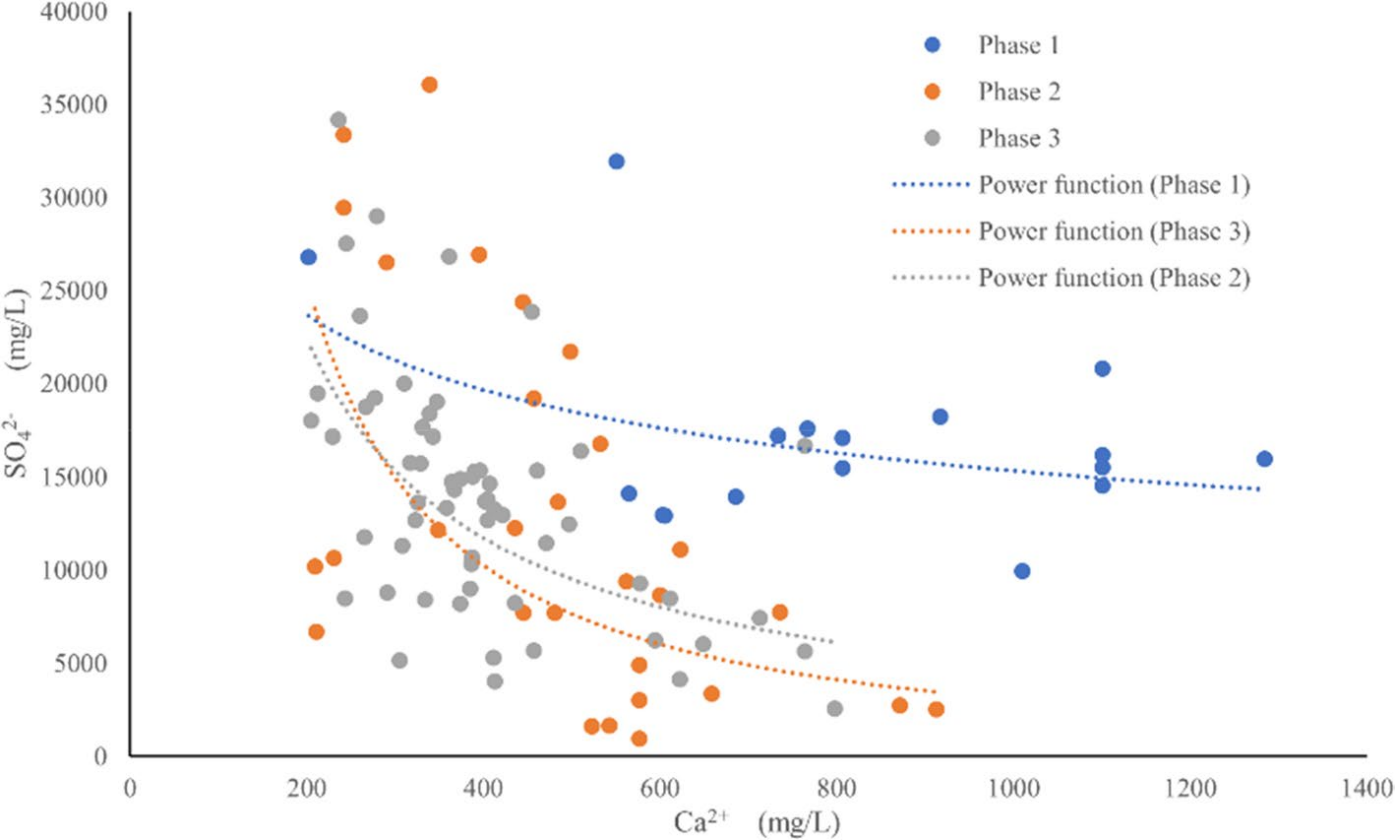
Scatter diagrams of SO_4_^2-^ and Ca^2+^ in brine at different stages.

The dissolution of salt minerals is always accompanied by two processes: dissolution and crystallization. In addition to dissolving mineral ions into the solution, there is also a process where ions in the solution are crystallized back to the surface of the mineral. According to the scatter plot of SO_4_^2-^ and Ca^2+^ in brine at different stages (Fig. 6), the content of SO_4_^2-^ is negatively correlated with Ca^2+^, and this relationship is more significant in the second and third stages. The average SO_4_^2-^ content in Mahai Lake is 34.30 g/L^24^, slightly higher than that of the primary underground brine. The mixing effect will cause SO_4_^2-^ and Ca^2+^ to exceed the solubility product, Ca^2+^ + SO_4_^2-^ → CaSO_4_↓, resulting in gypsum precipitation and Ca^2+^ content reduction.

### Cation exchange

Lu et al.^32^ believes that the weakly alkaline groundwater environment in Qaidam Basin is beneficial for cation exchange, where calcium ions with strong adsorption affinity replace the sodium ions adsorbed on the rock surface, resulting in a decrease in calcium ion concentration. The scale of cation exchange is determined by the adsorption capacity of the soil, while the specific surface area of the medium determines the adsorption capacity of the soil. Cation exchange adsorption is generally not observed in dense crystalline rocks^33^. The pH of the brine in the study area is about 7–8, which is a weakly alkaline environment. However, the rock formation with halogen medium in the study area is tight, and the specific surface area of rock and soil is small, making it more difficult for cation exchange to occur. In other words, the tight halogen storage medium of rock salt is more unfavorable to the occurrence of cation exchange.

Unlike the general freshwater storage media, the pores in the study area's brine storage media are mostly intercrystalline pores of salt minerals, and the original intercrystalline pore framework is susceptible to corrosion to form secondary pores. The dissolution of soluble salts in the medium during earlier stages of mining and extraction may have caused a change in the structure of the brine storage media, resulting in the loss of tight crystallization of salt crystals, which may have provided a possibility for cation exchange to occur. The Ca^2+^ in the brine has replaced the Na^+^ in the brine storage medium, causing a decrease in Ca^2+^. Cation exchange is also one of the reasons for the decrease in Ca^2+^ content in the brine.

### Changes in porosity

**Figure 7:**
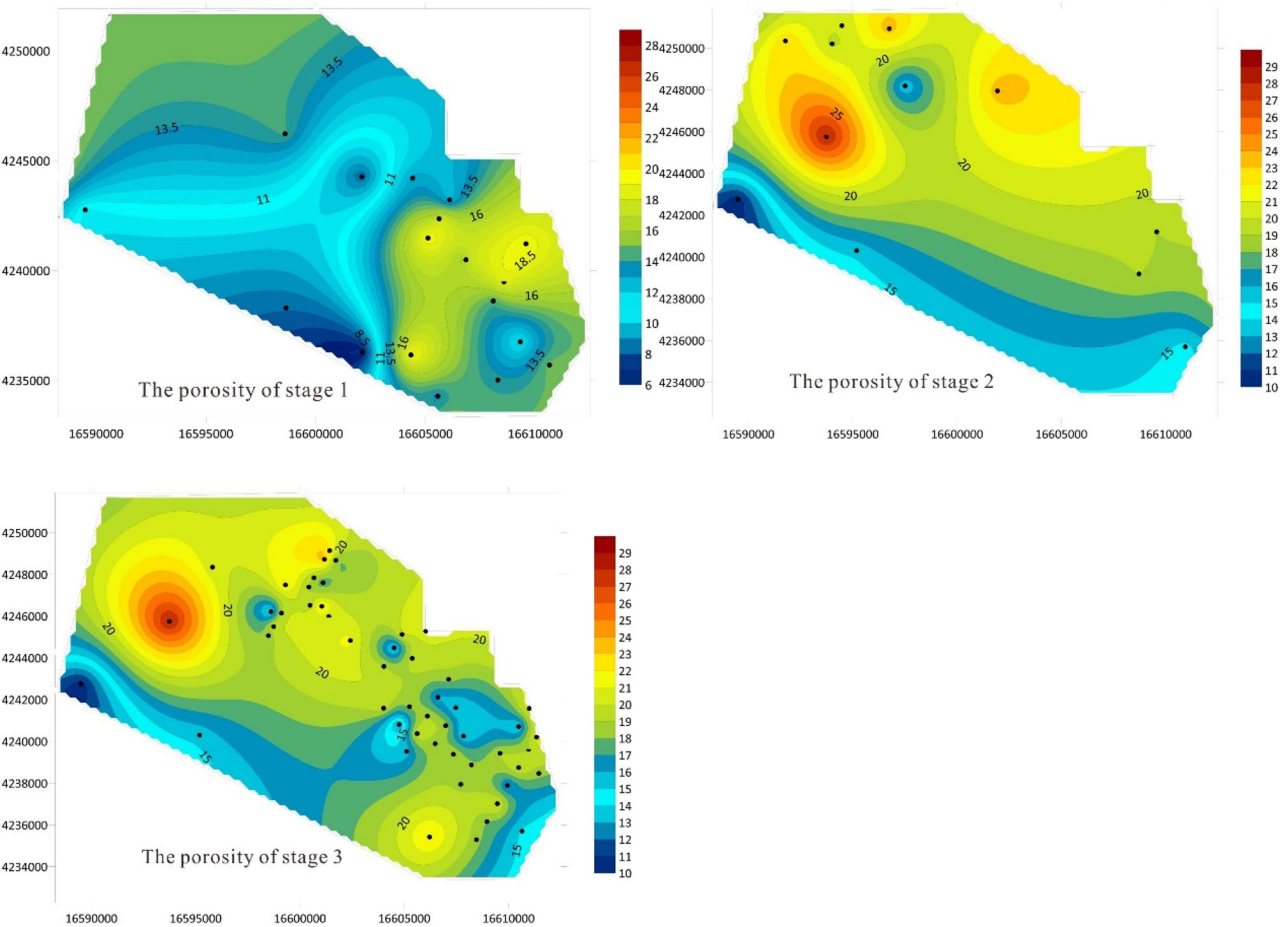
Distribution characteristics of halogen storage medium porosity at different stages.

The porosity of the brine storage medium is the storage space and transmission channel of underground brine and is the key index for the calculation and evaluation of brine resources. Therefore, analyzing the changes in porosity of the brine storage medium at different stages can effectively reflect changes in its structure. Figure 7 presents the distribution characteristics of porosity distribution in brine storage media at different stages. Different from the variation trend of Ca^2+^ concentration, the porosity of the study area showed an increasing trend on the whole. In the early stage of mining, the porosity in the middle part of the study area is relatively low, the porosity in half of the study area is 10.0%, and the porosity in the southeast part is locally higher, and the highest value is 20.0%. In the middle period of mining, the porosity of the study area improved on the whole, reaching 18% on average, and the highest porosity was 28.0% in the northwest. In the later stage of exploitation, the porosity of the study area is relatively high on the whole, and the porosity of more than half of the areas can reach 20.0%. When the lake water recharges into the aquifer, the original water–rock balance is destroyed, the rock salt crystal dissolves, the pore structure changes, and the formation porosity increases. With the increase of pores, the underground brine storage space and transmission channel become larger, and salt rock minerals are more likely to dissolve into the brine, thus further increasing the salinity of the brine.

## Conclusions

Different from the general freshwater storage medium, the brine storage medium in the Mahai Salt Lake is composed of soluble salt rocks. The dissolution of salt minerals during the process of brine extraction and leaching maintains a high mineralization, with an average mineralization exceeding 320.00 g/L. Na^+^ and Cl^−^ are the dominant ions in the brine, with relatively small changes in content during the mining process.

The Ca^2+^ content in the brine is relatively small, but the coefficient of variation changes significantly, indicating that its content is significantly influenced by environmental changes. The content of calcium ions decreased by 41.7% in the middle stage and 24.5% in the late stage compared with the early stage. The correlation between Ca^2+^ and mineralization changes significantly during different stages of mining, so Ca^2+^ can be used as a key index to study the salting-out effect of halogen storage.

There are three reasons for the decrease of Ca^2+^ content in brine: firstly, the dissolution of salt rock minerals maintains a high density and mineralization of secondary brine, but the low solubility of calcium-containing minerals leads to a relatively low Ca^2+^ content; secondly, the high SO_4_^2−^ content in the leaching lake water causes a mixing effect that exceeds the solubility product of Ca^2+^ and SO_4_^2−^, resulting in gypsum precipitation and precipitation; thirdly, the occurrence of cation exchange, where Ca^2+^ in the brine replaces Na^+^ in the brine storage medium, causes a decrease in Ca^2+^.

The formation porosity of Mahai Salt Lake shows an opposite trend to Ca^2+^. With the increase of pores, the underground brine storage space and transmission channel become larger, and salt rock minerals are more easily dissolved into the brine, thus further increasing its mineralization.

## Data Availability

The authors confirm that the data supporting the findings of this study are available within the article. The data that support the findings of this study are available on request from the corresponding author, [Shuya Hu], upon reasonable request.

## Abbreviations

Ca^2+^: Calcium ion
K^+^: Potassium ion
Na^+^: Sodium ion
Mg^2+^: Magnesium ion
EDTA: Edta titration method
TDS: Total dissolved solids
Cl^-^: Chloride ion
SO_4_^2-^: Sulfate ion
